# New sagittal abdominal diameter and transverse abdominal diameter based equations to estimate visceral fat area in type 2 diabetes patients

**DOI:** 10.1186/s12889-024-18659-8

**Published:** 2024-05-21

**Authors:** Chao Li, Liankun Zeng, Miaosheng Li, Kang Deng, Die Zhou, Rutao Liang, Xiaoshu Zhang, Zhihui Hu, Ai Luo, Chunling Chen, Qi Chen, Wenlong Wei, Wangen Li, Zhuoqing Hu

**Affiliations:** 1 Department of Endocrinology, the Second Affiliated Hospital of Guangzhou Medical University, Guangzhou, 510220 China; 2 Guangzhou Medical University, Guangzhou, 510220 China; 3 Guangdong Medical University, Zhanjiang, 524001 China; 4 Department of Endocrinology, the Fourth Affiliated Hospital of Guangzhou Medical University, Guangzhou, 511300 China; 5 Department of Joint surgery, Affiliated Southwest Hospital of Youjiang Medical University for Nationalities, Baise, 533099 China; 6 Baise People’s Hospital, Baise, 533099 China; 7 Affiliated Hospital of Guangdong Medical University, Zhanjiang, 524002 China; 8 Department of Clinical Laboratory, the Second Affiliated Hospital of Guangzhou Medical University, Guangzhou, 510220 China; 9 Department of Radiology, the Second Affiliated Hospital of Guangzhou Medical University, Guangzhou, 510220 China; 10 Guangzhou Red Cross Hospital(Guangzhou Red Cross Hospital of Jinan University), Guangzhou, 510240 China

**Keywords:** Visceral fat area (VFA), Sagittal abdominal diameter (SAD), Transverse abdominal diameter (TAD), Type 2 diabetes

## Abstract

**Objective:**

Computed Tomography (CT) or Magnetic Resonance Imaging (MRI) are considered gold standards for measuring visceral fat area (VFA). However, their relatively high prices and potential radiation exposure limit their widespread use in clinical practice and everyday life. Therefore, our study aims to develop a VFA estimated equation based on sagittal abdominal diameter (SAD) and transverse abdominal diameter (TAD) using anthropometric indexes. To the best of our knowledge, there have been limited studies investigating this aspect thus far.

**Methods:**

This study was designed as a cross-sectional, retrospective cohort survey. A total of 288 patients (167 males and 121 females) aged 18–80 with type 2 diabetes (T2D) were consecutively collected from a multicenter hospital, and VFA was measured by CT. Subsequently, variables highly correlated with VFA were screened through general linear correlation analysis. A stepwise regression analysis was then conducted to develop a VFA estimated equation. Discrepancies between the estimated and actual VFA values were assessed using the Bland-Altman method to validate the accuracy of the equation.

**Results:**

In the female T2D population, triglyceride (TG), SAD, TAD were found to be independently correlated with VFA; in the male T2D population, BMI, TG, SAD and TAD showed independent correlations with VFA. Among these variables, SAD exhibited the strongest correlation with VFA (*r* = 0.83 for females, *r* = 0.88 for males), followed by TAD (*r* = 0.69 for females, *r* = 0.79 for males). Based on these findings, a VFA estimated equation was developed for the T2D population: VFA (male) =-364.16 + 15.36*SAD + 0.77*TG + 9.41*TAD − 5.00*BMI (R^2^ = 0.75, adjusted R^2^ = 0.74); VFA(female)=-170.87 + 9.72*SAD-24.29*(TG^-1) + 3.93*TAD (R^2^ = 0.69, adjusted R^2^ = 0.68). Both models demonstrated a good fit. The Bland-Altman plot indicated a strong agreement between the actual VFA values and the estimated values, the mean differences were close to 0, and the majority of differences fell within the 95% confidence interval.

**Conclusions:**

In the T2D population, a VFA estimated equation is developed by incorporating SAD and TAD along with other measurement indices. This equation demonstrates a favorable estimated performance, suggesting to the development of novel and practical VFA estimation models in the future study.

## Introduction

With the evolution of people’s lifestyle, metabolic diseases such as overweight and obesity are increasing year by year. Among them, centripetal obesity has also garnered growing attention. Centripetal obesity, also referred to as central obesity or abdominal obesity, denotes the accumulation of excessive visceral fat. Visceral fat area serves as the gold standard for diagnosing centripetal obesity [1]. Visceral fat is a variety of bioactive adipokines and pro-inflammatory cytokines produced by very active organs [2]. However, an excess of visceral fat can lead to the onset of numerous metabolic diseases. Studies have consistently demonstrated that visceral fat poses a greater risk to the body than fat accumulated in other areas, making it a significant factor in the development of non-alcoholic fatty liver disease (NAFLD), diabetes, cerebrovascular disease (CVD), reflux esophagitis, and cancer [3].

Currently, CT and MRI serve as the gold standards for measuring visceral fat area [2]. Although MRI does not involve radiation, it is a costly procedure. Conversely, CT scans are relatively more affordable but still expensive and expose patients to radiation. Consequently, neither of these imaging methods can be widely accessible [4]. Apart from the above two methods, bio-impedance analysis (BIA) can also be utilized to measure visceral fat area. However, this approach requires professionals to operate and is cumbersome, limiting its use for anytime, anywhere measurements by patients. In addition, the detection of visceral fat through BIA remains costly and places a significant burden on many grassroots hospitals in China, hindering its widespread application. Therefore, it is essential to predict visceral fat area using simple and accurate methods. This would enable medical personnel to assess central obesity at any time during clinical practice and facilitate widespread use among the general population, encouraging self-monitoring of central obesity.

Some studies have attempted to estimate visceral fat area using waist circumference (WC) or other somatometric indexes, such as height, weight, neck circumference, and hip circumference. Among these indexes, WC is considered a simple and widely used method for evaluating centripetal obesity [5]. However, due to the influence of subcutaneous fat in the human body, it is impossible to accurately distinguish visceral fat from subcutaneous fat. The estimated equation for visceral fat area estimated by WC combined with other measurement indicators has a low fit, with an R^2^ value typically hovering around 0.5. Therefore, there is a need for further improvement in the estimation of estimated equation for visceral fat areas. In order to overcome these limitations and shortcomings, it is essential to identify better anthropometric indexes that can enhance the accuracy and precision of visceral fat area measurement. Compared with WC, BMI and other anthropometric indexes, SAD has higher reliability and accuracy in predicting visceral fat area, and also a strong correlation with the risks of diabetes and cardiovascular disease [6–9]. Although previous studies utilized SAD as an indicator to develop estimated equations for visceral fat area, they were often limited by small sample sizes and inadequate representativeness [10]. Therefore, we wonder whether visceral fat area can be estimated more accurately by employing SAD alone or in combination with other anthropometric indexes. The objective of this study is to explore the development of a estimated equation for visceral fat area and analyze the accuracy of its estimation using SAD alone or in conjunction with other anthropometric indexes.

## Method

### Research methods and Population

Our research was formulated as a cross-sectional, retrospective cohort investigation. Hospitalized patients aged 18–80 with T2D admitted to Department of Endocrinology, the Affiliated Hospital of Guangdong Medical University, the Second Affiliated Hospital of Guangzhou Medical University and the Fourth Affiliated Hospital of Guangzhou Medical University from January 2018 to December 2021 were consecutively collected. During their hospitalization, abdominal CT scans were performed. The following cases were excluded: (1) those currently undergoing systemic corticosteroid therapy; (2) those with hepatic and renal insufficiency; (3) hyperthyroidism or hypothyroidism; (4) severe disability and mental disorders; (5) pregnancy; (6) malignant tumors; (7) seroperitoneum or peritonitis. Following an overnight fast of 10 h, blood samples were obtained to assess blood glucose and lipid levels. Ethical approval for this study was obtained from the Ethics Committee of the hospital, and written informed consent was obtained from all participants.

In this study, hepatic and renal insufficiency in patients was defined as AST ≥ 120U/L, ALT ≥ 105U/L, and eGFR ≤ 30 ml/min/1.73m^2^. Hyperthyroidism was defined as FT3 > 6.08pmol/L, FT4 > 22pmol/L, and TSH < 0.27uIU/mL. Hypothyroidism was defined as FT4 < 12pmol/L and TSH > 4.2uIU/mL. The normal range for these indicators was as follows: AST = 13-40U/L, ALT = 0-35U/L, FT3 = 3.10–6.08 pmol/L, FT4 = 12.0–22.0 pmol/L, TSH = 0.27–4.2 uIU/mL.

### Measurements of anthropometric indexes and VFA

Following a complete fast of four hours, patients were positioned in a supine posture by a radiologist, and a CT scan was performed to locate the L4-L5 region. Subsequently, uniaxial tomography was conducted to acquire a CT image of the abdomen. The parameters used for this abdominal CT scan were: 120 kV, 270 mA, and a slice thickness of 10 mm [11]. Thereafter, relevant software was utilized to measure the VFA at the umbilical level of the abdomen.

SAD was measured using a portable sliding-type abdominal caliper. The measurement was taken from the upper arm of the pliers downwards to the midpoint between the bilateral iliac crests, in close proximity to the L4-L5 gap. During measurement, the examiner instructed the subjects to inhale and exhale gently, and TAD was measured at the horizontal gap between L4 and L5, perpendicular to SAD. BMI = body mass(kg)/ height(m)^2^. All the aforementioned indicators were obtained through three measurements taken and then averaged.

### Serological test

After fasting for 8–10 h, all patients underwent measurements of fasting blood glucose (FPG), fasting C-peptide (FCP), glycosylated hemoglobin A1c (HbA1c), total cholesterol (TC), triglycerides (TG), high-density lipoprotein cholesterol (HDL-C), and low-density lipoprotein cholesterol (LDL-C) in the early morning following admission. FPG levels were measured using the glucose oxidase method, FCP levels were measured via chemiluminescent immunoassay, and blood lipid profiles were measured using an automatic analyzer for all subjects. HbA1c levels were determined using high-pressure liquid chromatography.

### Statistical analysis

All statistical analyses were conducted using Empower® (www.empowerstats.com, X&Y Solutions, Inc., Boston, MA) and R software (http://www.r-project.org). A significance level of *P* ≤ 0.05 was considered to be statistically significant. Continuous variables following a normal distribution were presented as mean ± standard deviation and compared between groups using a T-test. Continuous variables not following a normal distribution were expressed as median (Min-Max) and compared between groups using a Wilcoxon rank sum test. Enumeration or categorical data were presented as frequency or percentage (%) and compared between groups using the chi-square test. The correlation between different variables and VFA was assessed by Pearson or Spearman general linear correlation analysis. VFA-related variables were sequentially imported into the multiple linear regression equation for screening, identifying predictors independently related to VFA. Subsequently, a VFA estimated equation was built using a generalized linear model regression equation. Ultimately, the accuracy and consistency of the equation were verified through reliability analysis and Bland-Altman plot analysis.

## Results

### General Clinical features of T2D patients

A total of 288 patients aged 18–80 with T2D were enrolled in this study. There were 167 males and 121 females, and they were grouped by sex. Detailed characteristics of the general population in the two groups are presented in Table 1. It can be seen that apart from age, no significant differences were observed between the two groups in terms of other variables.

**Table 1:** Clinical Features of T2D Patients

Sex	Female	Male	*P*-value
Number of cases (N)	121	167	\
Age (yrs)	61.50 (11.44)	58.37 (12.41)	0.03
BMI (kg/m^2^)	23.39 (3.60)	23.01 (3.24)	0.37
FCP (µg/L)	2.29 (0.15–7.93)	1.83 (0.01–11.56)	0.55
HbA1c (%)	8.20 (5.30–17.00)	9.30 (5.00–19.00)	0.62
FPG (mmol/L)	7.29 (2.74–21.98)	7.16 (2.59–21.14)	0.94
TC (mmol/L)	4.69 (1.42–9.96)	4.63 (1.92–18.66)	0.95
TG (mmol/L)	1.46 (0.49–13.11)	1.42 (0.38–55.97)	0.29
HDL-C (mmol/L)	1.05 (0.22–2.16)	0.99 (0.19–3.16)	0.44
LDL-C (mmol/L)	2.79 (0.27–6.70)	2.77 (0.29–8.54)	0.92
VFA (cm^2^)	126.24 (21.56-270.52)	133.27 (16.54-345.76)	0.33
SAD (cm)	20.14 (3.05)	21.30 (3.22)	0.00
TAD (cm)	30.73 (2.89)	30.72 (2.57)	0.96

BMI: Body Mass Index; FCP: Fasting C-Peptide; HbA1c: Glycosylated Hemoglobin A1c; FPG: Fasting Blood Glucose; TC: Total Cholesterol; TG: Triglyceride; HDL-C: High-Density Lipoprotein Cholesterol; LDL-C: Low-Density Lipoprotein Cholesterol; VFA: Visceral Fat Area; SAD: Sagittal Abdominal Diameter; TAD: Transverse Abdominal Diameter

### General Linear Correlation Analysis

Table 2 displays the coefficients associated with VFA-related variables. In females, BMI (*r* = 0.63, *P* < 0.01), FCP (*r* = 0.27, *P* < 0.05), HDL-C (*r*=-0.20, *P* < 0.05), TG (*r* = 0.33, *P* < 0.01), TAD (*r* = 0.69, *P* < 0.01), SAD (*r* = 0.83, *P* < 0.01) were strongly correlated with VFA. In males, BMI (*r* = 0.68, *P* < 0.01), FCP (*r* = 0.58, *P* < 0.01), TG (*r* = 0.41, *P* < 0.01), HDL-C (*r*=-0.26, *P* < 0.01), TAD (*r* = 0.79, *P* < 0.01), SAD (*r* = 0.88, *P* < 0.01) were strongly correlated with VFA. Furthermore, scatter plots were generated for these six variables against VFA, illustrating a clear linear relationship between them.

**Table 2:** The Correlation Coefficient of VFA with Demographic and Anthropometric Variables

Var1	Var2	Correlation (Female)	*P*-value	Correlation (Male)	*P*-value	
Age	**VFA**	0.16	0.08	0.15	0.06	
BMI	**VFA**	0.63	0.00	0.68	0.00	
FCP	**VFA**	0.27	0.01	0.58	0.00	
HbA1c	**VFA**	-0.14	0.15	-0.14	0.10	
TC	**VFA**	0.06	0.50	0.15	0.06	
TG	**VFA**	0.33	0.00	0.41	0.00	
HDL-C	**VFA**	-0.20	0.04	-0.26	0.00	
FPG	**VFA**	0.03	0.79	-0.04	0.67	
TAD	**VFA**	0.69	0.00	0.79	0.00	
SAD	**VFA**	0.83	0.00	0.88	0.00	
LDL-C	**VFA**	0.04	0.69	0.03	0.67	

BMI: Body Mass Index; FCP: Fasting C-Peptide; HbA1c: Glycosylated Hemoglobin A1c; FPG: Fasting Blood Glucose; TC: Total Cholesterol; TG: Triglyceride; HDL-C: High-Density Lipoprotein Cholesterol; LDL-C: Low-Density Lipoprotein Cholesterol; VFA: Visceral Fat Area; SAD: Sagittal Abdominal Diameter; TAD: Transverse Abdominal Diameter

### Screening of Stepwise Regression Analysis

**Fig. 1:**
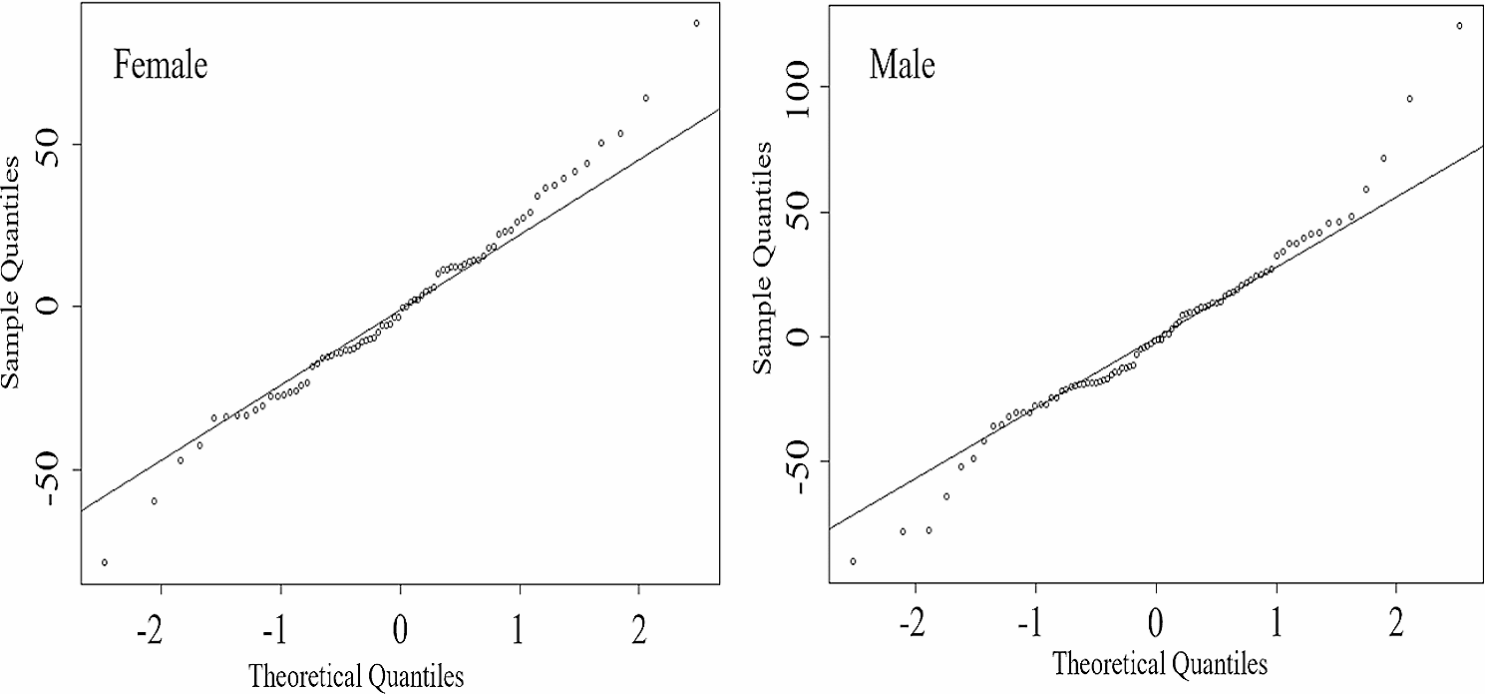
QQ Plot of Residuals for Male and Female VFA Estimation Equations

A stepwise regression analysis was conducted on SAD, TAD, BMI, TG, FCP and HDL-C. The results revealed that in the female population, TG, SAD, and TAD were independently and significantly correlated with VFA. In the male population, BMI, TG, SAD, and TAD were independently and significantly correlated with VFA (refer to Table 3 for details). By employing the aforementioned indexes as independent variables and VFA as the dependent variable, we developed a VFA estimated model using stepwise regression analysis. VFA(Female)=-170.87 + 9.72*SAD − 24.29*(TG^-1) + 3.93*TAD (R^2^ = 0.69, adjusted R^2^ = 0.68);VFA(Male)=-364.16 + 15.36*SAD + 0.77* TG + 9.41*TAD − 5.00*BMI (R^2^ = 0.75, adjusted R^2^ = 0.74). These two models demonstrated a good fit, with variance inflation factors of the variables all below 4, indicating the absence of collinearity among the independent variables. Furthermore, Fig. 1 showed that the residuals of regression model were distributed around a line, indicating a constant dispersion degree and normal distribution of residuals.

**Table 3:** The Establishment of New Equations for Male and Female Groups Respectively

Sex	Equation	*R* ^2^	Adjusted *R*^2^	Variables	95%CI	*P*-value	VIF
Female	-170.87 + 9.72*SAD − 24.29*(TG^-1) + 3.93*TAD	0.69	0.68	SAD	7.58,12.97	0.00	2.4
				TG	-0.27,6.23	0.08	1.2
				TAD	0.98,6.34	0.01	2.2
				Constant	-252.95, -140.12	0.00	/
Male	-364.16 + 15.36*SAD + 0.77*TG + 9.41*TAD − 5.00*BMI	0.75	0.74	SAD	12.56,20.93	0.00	5.0
				TAD	4.40,13.49	0.00	4.0
				BMI	-9.09, -2.14	0.00	4.0
				TG	-2.70,4.28	0.66	1.1
				Constant	-445.00, -286.60	0.00	/

BMI: Body Mass Index; TG: Triglyceride; SAD: Sagittal Abdominal Diameter; TAD: Transverse Abdominal Diameter

### Verification of Stepwise multiple Linear regression equation Model

**Fig. 2:**
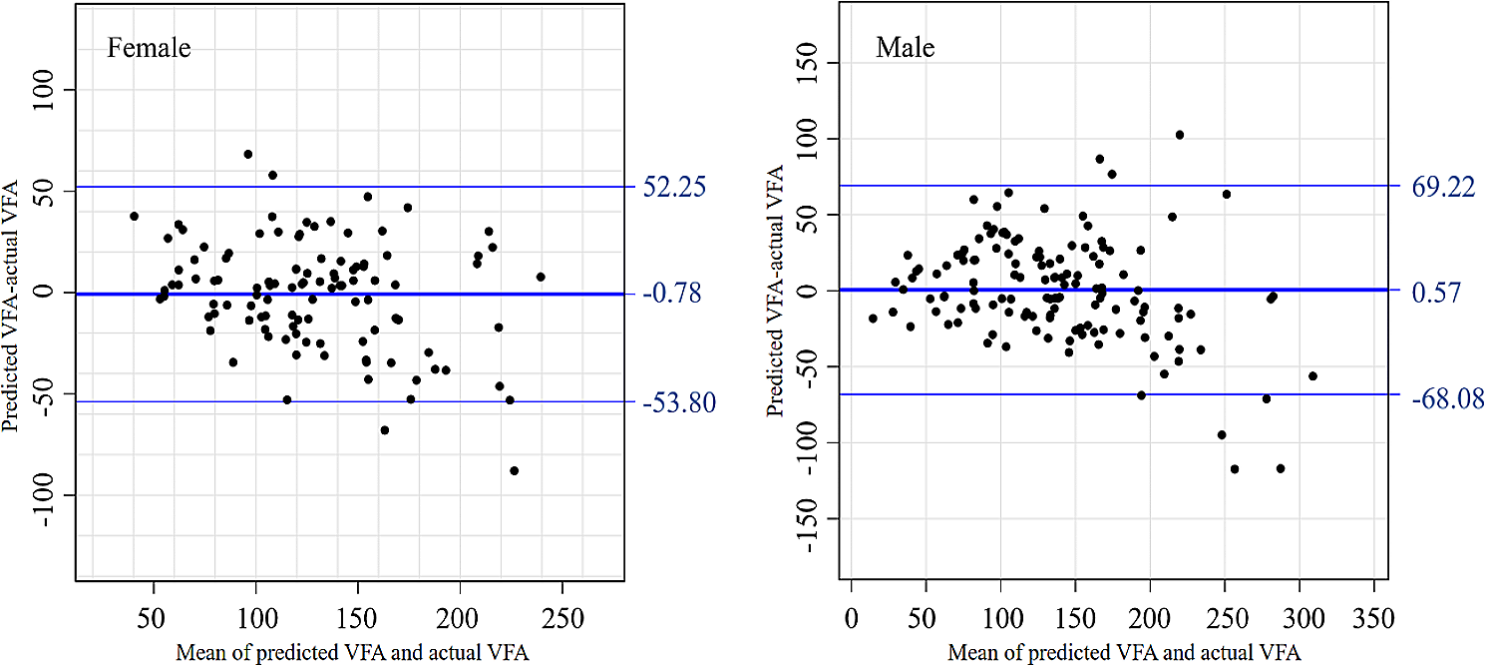
The Bland-Altman Plot of Actual VFA and Predicted VFA.

Quantitative measurement method was adopted to compare the difference between the predicted values and the actual values of visceral fat obtained by the estimation model. The Bland-Altman plot revealed a strong agreement between predicted and actual values for both males and females (refer to Fig. 2). The mean difference between the predicted and the actual VFA values was close to 0, with the majority of differences falling within the 95% confidence interval.

Therefore, the aforementioned results demonstrated that the estimated equation obtained by constructing a block with the estimated model had robust estimation power and high value in clinical practice. In scenarios where CT or MRI examinations were unfeasible for certain patients or medical facilities, clinicians can assess BMI, TG, SAD and TAD of each patient and substitute them into the estimated equation, to predict VFA value in an accurate, portable and quick manner.

The upper and lower horizontal solid lines in the figure represented the 95% limits of agreement, while the middle horizontal solid line represented the average difference value. The horizontal dotted line indicated the point where the average difference value equaled zero.

## Discussion

Excessive accumulation of abdominal visceral fat can lead to centripetal obesity, triggering a range of severe metabolic and cardiovascular conditions. Currently, the assessment of VFA using CT, MRI, or BIA is not widely feasible in clinical settings, making it challenging for the general population to monitor their obesity levels whenever and wherever possible, especially in individuals with T2D. Many T2D patients with normal weight and BMI may exhibit centripetal obesity without recognizing it. The absence of intervention and monitoring for long-term concentric obesity significantly can contribute to the onset and progression of associated complications. Thus, it is crucial to accurately and simply estimate VFA [12–14].

Sex hormones are an important factor affecting the distribution and area of fat, so in this study, we analyzed data and estimated VFA in male and female populations respectively [15]. It was found through correlation analysis that SAD, TAD, BMI, TG, FCP and HDL-C were strongly correlated with VFA in both groups. By screening using the stepwise multiple regression equation, we found that in the female T2D population, TG, SAD and TAD were significantly and independently correlated with VFA; while in the male T2D population, BMI, TG and SAD were significantly and independently correlated with VFA. Finally, the above variables were utilized to developed a VFA estimated model and it was observed that these two equations had good fit and accuracy (R^2^ = 0.69, adjusted R^2^ = 0.68 for females; R^2^ = 0.75, adjusted R^2^ = 0.74 for males). The predicted VFA values were compared with the actual VFA values, revealing a significant and high level of agreement, with the majority falling within the 95% confidence interval, and the mean difference being close to zero. The relationship between the observed variables X and the observed outcome Y is mostly not simply linear, but rather exhibits a curved correlation. As VFA continues to increase, it may exceed the capacity of the estimating variables, necessitating the inclusion of more variables to accurately represent the true VFA value. Consequently, the estimated VFA in our study is lower than the actual VFA.

In general, men tend to exhibit a lower body fat percentage and a higher visceral fat percentage, resulting in an apple-shaped physique, while women typically exhibit the opposite, presenting a pear-shaped body. The table in our research findings highlights the differences in fat distribution between males and females, and in the variables utilized for estimating VFA, SAD demonstrated significant variations across genders, which may underscore the need for additional BMI variables in estimating visceral fat area among males with T2D. Similar findings have been noted in previous studies, such as those by Brundavani V, et al., who also observed that males required supplementary BMI data to improve the accuracy of VFA estimation [16].

WC is the most widely used as a simple indicator for diagnosing centripetal obesity. In the Chinese population, the cut-off value for diagnosing centripetal obesity is ≥ 90 cm for males and ≥ 85 cm for females [17, 18]. However, due to the influence of subcutaneous fat area (SFA), WC cannot distinguish between visceral fat and subcutaneous fat. Although the VFA estimated equation has been established by incorporating WC and other anthropometric indexes, such as neck circumference and BMI, it has a small R value, so the fitting degree remains to be enhanced [9, 16, 19, 20], indicating the need for further improvement in the estimated equation. At the L4-L5 gap, the maximum anterior-posterior width is SAD, and the maximum span width is TAD [21]. SAD is a novel, popular and robust predictor for VFA, which can roughly illustrate the distribution of abdominal fat and is easy to measure and access. Studies have shown that SAD exhibits a stronger correlation with VFA than WC and is closely associated with the occurrence of metabolic diseases and cardiovascular events [7, 9, 22]. As far as we know, our work is the second study to build VFA estimated equation by using SAD in combination with other anthropometric indexes. Despite the small sample sizes of previous studies, it was still demonstrated that SAD was an excellent predictor for VFA [10]. While TAD was closely linked with SFA, it was also strongly correlated with VFA and accurately predicts VFA [21, 23]. Consistent with prior findings, our results also demonstrated that SAD had the highest correlation coefficient with VFA, followed by TAD, both surpassing BMI. Hence, the utilization of SAD, TAD, and other anthropometric indexes in developing a estimated equation and estimate VFA in this study proves to be more superior and advantageous for monitoring and assessing obesity in T2D patients.

According to the guidelines in many countries, elevated fasting TG is deemed as one of the independent conditions for diagnosing metabolic syndrome [17, 24], highlighting the closer association of TG with metabolic diseases compared with other blood lipid components. Our findings demonstrate that TG is significantly and independently correlated with VFA, emerging as a crucial index for VFA estimation. In patients with non-diabetic obesity, serum TG was also found to be positively and independently correlated with VFA, serving as a key predictor of VFA [25]. In the population with normal weight or BMI, concentric obesity is identified as a significant risk factor for cardiovascular disease, showing the highest correlation with elevated TG levels [26]. Research has suggested that concentric obesity is linked to increased intracellular storage of TG in fat-free masses such as muscles, liver, and pancreatic β cells. TG is accompanied by high concentration of cytosolic long-chain acyl-CoA esters in cytoplasm, which promotes the generation of oxygen free radicals in endothelial cells, diminishes the scavenging ability of oxygen free radicals by β cells, and results in endothelial dysfunction and progressive failure of β cells. This sheds light on the close relationship between TG and cardiovascular risk events, as well as T2D from the perspective of pathological mechanism [27].

There are certain limitations in this study. Firstly, due to constraints in manpower and funding, the relatively small sample size selected for this study may potentially impact the accuracy of VFA estimation. What’s more, the proposed equation is only applicable to T2DM patients who have undergone abdominal CT scans and not to (1) those currently undergoing systemic corticosteroid therapy; (2) those with severe hepatic and renal insufficiency; (3) hyperthyroidism or hypothyroidism; (4) severe disability and mental disorders; (5) pregnancy; (6) malignant tumors; (7) seroperitoneum or peritonitis. Furthermore, as the number of minors with T2D is limited, primarily affecting middle-aged or elderly patients, the selected participants in this study were all adults. For this reason, the established VFA estimated model is not suitable for minors under the age of 18, and the equation requires further refinement and exploration. Lastly, despite the exclusion of patients with tumors, abdominal effusion, and peritoneal inflammation, its applicability to patients who have not undergone abdominal CT scans remains uncertain. Last but not least, the aim of this research is to utilize anthropometric indicators to estimate VFA and to devise additional equations for estimating VFA in forthcoming studies. In this investigation, VFA measurement was conducted using a 10 mm slice thickness during abdominal CT scanning. There is a possibility that VFA measured with a 10 mm slice thickness may not be appropriate for the diagnosis of central obesity. Hence, prudence is advised when utilizing the estimated VFA values from this study for diagnosing central obesity, and it is imperative for future research to ascertain the suitable CT slice thickness for detecting VFA values in the diagnosis of central obesity.

Taken together, in this study, SAD, TAD and TG are found to be independently correlated with VFA in T2D population, with SAD showing the closest correlation, suggesting that SAD may be superior in identifying central obesity and predicting metabolic disorders. Future research could explore comparing SAD with alternative indicators like WC, BMI, and so forth, to diagnose central obesity and predict susceptibility to metabolic diseases. This endeavor aims to identify simpler, more convenient, and accurate somatometric indicators for facilitating daily self-assessment of central obesity and for use in extensive epidemiological investigations. For the first time, we have utilized SAD and TAD in combination with TG or BMI to establish a VFA estimated equation, and demonstrated that the model had a high fitting degree, and the predicted VFA value was in good agreement with the actual value. This model can serve as an important tool for clinical practice or daily self-monitoring of concentric obesity in T2D patients, contribute to the management of long-term diabetes complications. Nevertheless, as mentioned, this study has certain limitations. In the future, it will be crucial to increase the sample size, diversify the study population, and validate the accuracy and effectiveness of SAD in combination with TAD to assess VFA.

## Data Availability

The datasets generated and analyzed during the current study are not publicly available due privacy and ethical restrictions but are available from the corresponding author on reasonable request.
